# VISOR-NET: Visibility Estimation Based on Deep Ordinal Relative Learning under Discrete-Level Labels

**DOI:** 10.3390/s22166227

**Published:** 2022-08-19

**Authors:** Lina Xun, Huichao Zhang, Qing Yan, Qi Wu, Jun Zhang

**Affiliations:** 1 The Key Laboratory of Intelligent Computing and Signal Processing of Ministry of Education, School of Electrical Engineering and Automation, Anhui University, Hefei 230601, China; 2 School of Artificial Intelligence, Anhui University, Hefei 230601, China

**Keywords:** deep learning, ordinal regression, relative learning, visibility estimation

## Abstract

This paper proposes a novel end-to-end pipeline that uses the ordinal information and relative relation of images for visibility estimation (VISOR-NET). By encoding ordinal information into a set of relatively ordered image pairs, VISOR-NET can learn a global ranking function effectively. Due to the lack of real scenes or continuous labels in public foggy datasets, we collect a large-scale dataset that we term Foggy Highway Visibility Images (FHVI), which are taken from real surveillance scenes, and synthesize an INDoor Foggy images dataset (INDF) with continuous annotation. This work measures the estimation effectiveness on two public datasets and our FHVI dataset as a classification task and then on the INDF dataset as a regression task. Comprehensive experiments with existing deep-learning methods demonstrate the performance of the proposed method in terms of estimation accuracy, the convergence rate, model stability, and data requirements. Moreover, this method can extend inter-level visibility estimation to intra-level visibility estimation and can realize approximate regression estimation under discrete-level labels.

## 1. Introduction

Visibility is a complex phenomenon that is affected by emissions, air pollutants, and other factors, including sunlight, humidity, temperature, and time. As a human-perceived concept, it is usually referred to as the distance of ‘an object being just visible’. The World Meteorological Organization (WMO) defines it as the longest distance at which a black object of suitable dimensions, located on or near the ground, can be seen and readily identified when observed against the horizon [1].

Fog, a very common atmospheric phenomenon with horizontal visibility of less than 1000 m, influences human society in many ways [2]. Usually, atmospheric visibility is measured by two major approaches: optical sensor-based and visual performance-based approaches. The former measures every possible atmospheric parameter, such as light scatter, air light, and light absorption, to derive visibility from a small part of the atmosphere, so the accuracy is limited. The latter relies on professional meteorologists. Due to high costs and complexity, the sensor-based meters installed by weather stations are not geographically extensive. As for visual observations by humans, they may be subjective, and they are easily affected by experience and the observation environment. 

Fortunately, many video-surveillance cameras are widely deployed in public and private places. Estimating atmospheric visibility from a surveillance image has great value in meteorology, public transportation, and many other fields and has caught many researchers’ attention [3]. Some previous image-based methods require the detection of a specified target or the use of auxiliary equipment [4]. Since the additional information or extra device is not equipped in normal vision systems, the applications of these algorithms are limited. Therefore, single-image-based visibility estimation has been developed in recent years [5]. 

Some simple image features, including the brightness, contrast between a target and background [6,7], and gray-scale level [8], are already used to estimate visibility. However, these basic image features are sensitive to illumination variations. Fourier transform-combined Sobel operations [9] are adopted to extract global features of the image to overcome illumination variations. Koschmieder’s law-based methods have been investigated extensively to estimate visibility, which describes the relation between transmission and atmospheric light and how haze or fog impacts the observed image [9,10,11,12]. However, these techniques need prior knowledge or manual settings [13] to estimate the atmospheric light and transmission map, and thus face many challenges, such as light sources and absorption affecting the accuracy of the visibility estimation. In addition, Koschmieder’s law-based methods always build a model by assuming that the atmosphere is uniform, which is rare in real-world situations. Therefore, a learning-based approach that can be generally applied or adapted to different scenes is needed.

Inspired by the successful application of convolutional neural networks (CNN) in many computer vision tasks, researchers have turned their attention to learning-based approaches to visibility problems. Based on annotated image data, a CNN model was trained to obtain the final classification of visibility [14]. However, it is still a very challenging problem to specify the absolute visibility from a single image accurately, even for human beings. Conversely, humans can easily specify relative relations between two images with different visibility levels. Therefore, a relative CNN-RNN was proposed to find the relative features from paired images [15], which first proved the effectiveness of relative relations in visibility estimation.

Currently, there are two challenges to overcome for deep learning-based methods. One of them is that the available high-quality datasets are insufficient. Deep learning is a data-driven approach, and its performance heavily depends on the dataset size and annotation quality. However, only two synthetic image-classification datasets, Foggy ROad Sign Images (FROSI) [16] and Extended Foggy Road Image Database (ExFRIDA) [17], have been released recently. A large-scale dataset of annotated images collected from real scenes is still unavailable. Another challenge is that estimating the visibility level from the image is difficult when only using an ordinary classification model. Because of the ordinal information hidden in the data, visibility estimation is an intermediate task between regression and classification. Generally, visibility estimation can be viewed as a typical regression problem with many continuous labels, and the ordinal information of visibility among all images is more valuable for model training. In real scenes, the continuous visibility value for surveillance images is very difficult to acquire. However, the level labels for visibility contain intrinsic ordinal information. If the ordinal information among all images can be used to aid visibility estimation, the model performance is improved.

In this paper, we address these two challenges by collecting a large-scale dataset named Foggy Highway Visibility Images (FHVI) from real surveillance scenes and proposing VISOR-NET, a novel end-to-end pipeline, which is different from the existing deep learning-based classification or regression methods. Extensive experiments show that VISOR-NET can achieve better performance than the current state-of-the-art models in all visibility datasets.

The main contributions of this paper are summarized as follows:

(1) A novel end-to-end pipeline VISOR-NET is proposed for visibility estimation with ordinal relative learning, which combines ordinal regression with the relative features of images to map relative visibility values. Compared to the existing algorithms, the proposed method can achieve better performance with fewer image data in a short training time.
(2) A large-scale dataset, FHVI, taken from real surveillance data is collected. The visibility label of each image is annotated and manually checked by meteorological staff with reference to a professional visibility meter. This dataset will benefit further visibility estimation research.
(3) After adding a small number of continuous labels to training images as anchors, the relative values of VISOR-NET can be mapped to the real visibility. Regression experimental results demonstrate that the proposed VISOR-NET can obtain a satisfactory global regression function for visibility estimation under discrete class labels.

## 2. Related Work

Visibility estimation techniques can be divided into two broad categories: data-driven and statistical methods. Considering that our main concern is data-driven methods, we give a brief introduction to them here. Other related techniques, including ordinal regression and relative learning, are also discussed here.

### 2.1. Data-Driven Methods

As early as the 1990s, a simple feed-forward neural network [4] was proposed to improve short-range visibility forecasts. A similar study was also conducted to map the nonlinear relation between visibility and multiple metrological features [18]. However, these studies only use metrological data because extensive image datasets for visibility estimation were unavailable at that time. Recently, Chaabani et al. used an artificial neural network (ANN) [19] to estimate the visibility distance under foggy weather conditions from camera images. 

However, such a simple ANN-based model cannot handle complicated real-world situations. Therefore, deep learning-based methods have been proposed. Li et al. first employed a CNN [20] to estimate the visibility distance from webcam images and a pre-trained AlexNet [21] was used to extract the features for classification. Giyenko also built a shallow CNN (SCNN) [22] containing three convolutional layers to conduct visibility detection on camera images. Akmaljon Palvanov et al. proposed an improved VISibility CNN-based network (VisNet) [23], where a fast Fourier transform (FFT) algorithm and a high-pass filter were used to filter the original image into two images of the same size, and these images combined with the original image were input in an integrated CNN to obtain the final visibility classification. In essence, VisNet is a data augmentation method. In contrast to other methods, You et al. [15] proposed a relative CNN-RNN to estimate the relative atmospheric visibility from outdoor images, where the CNN-RNN network was used to extract the relative features and the final classification task was conducted with a support vector machine (SVM). However, this method is not end-to-end and does not take full advantage of the potential capacity because it still ignores the ordinal information of images.

### 2.2. Ordinal Regression

Ordinal regression is a technique for predicting the ordinal relationship from a set of independent features, and it is widely used in age estimation [14], face recognition [24], and monocular depth estimation [25]. Such as sensory grading of symptoms (no pain/slight pain/relatively pain/severe pain), age grading (1–18/…/60–100 years old), etc. There is a relative ranking among different values in the range of variables, but the differences between grades are not equal. For example, the age difference between young people and children is not necessarily equal to the age difference between the old and the middle age. Generally, there are two main types of approaches to solving ordinal problems. One is converting the ordinal regression problem to a m−1 binary classification problem, and the k-th classifier is trained to predict the probability of $y_{t}>k$ for the labeled instance $\left(x_{t},y_{t}\right)$. Frank et al. [26] and Li et al. [27] put this idea into practice using several decision trees or a REDuction SVM (RED-SVM). The other approach can be informally described as transforming the ordinal regression to a regular regression problem, where the ordinal information of classes can be preserved. It is also named a threshold approach in [28], and its goal is to learn the latent function and the boundaries of the intervals between ranks. Kramer et al. [29] investigated a regression-tree learner by mapping the ordinal scale to real numeric values. The original SVM [30], kernel-discriminant analysis (KDA) [31], and learning-vector quantization (LVQ) [32] were extended by a rank constraint to be suited for ordinal regression.

Recently, deep convolutional neural networks have been applied to this problem. Niu et al. [14] transformed the ordinal problem into a series of binary classification problems using a Multiple Output CNN (MOCNN) for age estimation. Fu et al. [25] introduced a spacing increasing discretization (SID) strategy to discretize depth and transform depth estimation into an ordinal regression problem, which achieved a much higher accuracy and faster convergence. Liu et al. [33] introduced the idea of large-margin deep neural networks and proposed a Convolutional Neural Network with Pairwise regularization for Ordinal Regression (CNNPOR). It is a weighted combination of the softmax logistic regression loss and the pairwise constraint from adjacent ranks. The former is used to distinguish different categories of examples and the latter maps the instances to a line with a large margin, which sets the minimum distance between the examples of adjacent rank as 1.

The first type, similar to a MOCNN, can be seen as an enhanced classification method combined with additional ordinal information by which a linear projection cannot be obtained. The main drawback of the second type (e.g., a CNNPOR) is that it cannot build a satisfying global function since the pairwise constraint from adjacent ranks is limited. In contrast to these two approaches, the proposed VISOR-NET learns a line by fully using the ordinal constraint from the dataset without a large margin setting.

### 2.3. Relative Attributes Learning

First, download relative attributes learning is an approach that finds a ranking function for each attribute using relative similarity constrains such as pairs of examples. Parikh and Grauman [34] proposed a method to compare the strength of a certain attribute between data and employed SVM to learn the ranking functions and predict the relative relationships for novel images. The Ranking SVM [35] has been extended to learn relative parts using local parts features that are shared across different categories. Li et al. [36] converted the decision tree into a relative tree and created a relative forest algorithm for nonlinear ranking function learning.

As traditional machine learning algorithms use handcrafted visual features to learn a ranking function, deep learning is also employed in relative attribute learning. The deep relative attributes (DRA) method [37] can learn the visual features and ranking function jointly using an end-to-end framework. The deep relative distance learning (DRDL) method [38] projects raw vehicle images into metric space and measures the similarity of two arbitrary vehicles using the relative Euclidean distance. Compared with traditional relative attribute learning methods, deep learning-based methods have significantly improved the accuracy of many research fields.

Intuitively, the visibility of images can also be considered a relative attribute. When fog is thicker, the visibility attribute is lower. Compared with the threshold approaches in ordinal regression, relative learning focuses on the intensity of some attributes between examples and does not need a pre-defined margin between ranks. Therefore, a linear projection obtained in this way is more natural. In general, relative learning does not consider global ordinal information from the whole dataset and the comparison between examples is random. Therefore, the training process is not stable or efficient.

## 3. The Proposed Method

**Figure 1:**
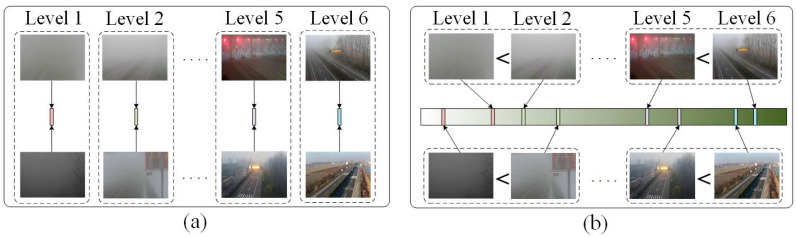
Demonstration diagram of the ordinary classification (**a**) and the proposed ordinal relative estimation (**b**). The green axis shows the visibility, which increases from left to right. In (**a**), foggy images are inducted into corresponding levels. In (**b**), the method compares the strength of visibility in paired images and projects on the visibility axis with ordinal constraints.

VISOR-NET is a novel ordinal regression method. In this paper, we encode the ordinal information into a series of paired images with relative order in each image batch. The proposed VISOR-NET learns a global rank function to quantify all the ordinal relative relations of images. Figure 1 shows a demonstration diagram of the ordinary classification (a) and the proposed ordinal relative estimation (b). In contrast to classification methods that map the image features directly to the category labels, VISOR-NET learns an estimation function f(.) for every image based on the intrinsic constraint of ordinal relative relations and encourages the keeping of both inter-class and intra-class differences. Even in the same category label, the fog concentration of different foggy images is still different. Therefore, our proposed method is to predict the continuous fog level of foggy images by ordinal relative learning. In contrast to the regression method, our estimation process does not require the use of continuous labels.

### 3.1. Model Architecture

**Figure 2:**
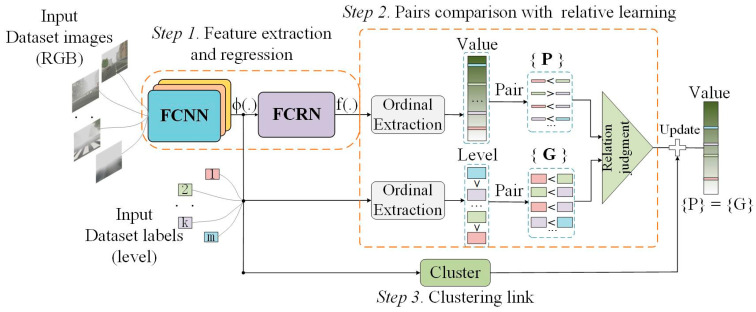
Schematic diagram of the proposed VISOR-NET with batch images.

The proposed VISOR-NET consists of three components, and the whole pipeline is shown in Figure 2. The first component is the feature extraction and regression module (FERM). In this step, a foggy convolutional neural network (FCNN) and a fully connected regression network (FCRN) are used as a feature extractor Φ(.) and an estimation function f(.), respectively. The second component is a pairs comparison with relative learning, where the ordinal relationships within one batch are encoded into thousands of paired relative ordering sets G to constrain the relative learning and make the estimation outputs consistent with the original ordinal. The P and G in Figure 2 mean predictions and ground truth, respectively. And the relation judgment means that the ordinal relationship between the predictions and the ordinal relationship between the ground truth should be consistent. Specifically, it refers to the logistic-like loss between paired images in Section 3.3. The third component is an optional clustering link, where a lightweight clustering loss is added to assist convergence, which constrains the distribution range of values to facilitate the classification task.

It must be pointed out here that the outputs of the VISOR-NET are continuous values. In visibility datasets where only level labels are provided, we use the K-nearest neighbor algorithm (KNN) with majority voting to predict the final visibility level.

### 3.2. Feature Extraction Regression Module (FERM)

**Figure 3:**
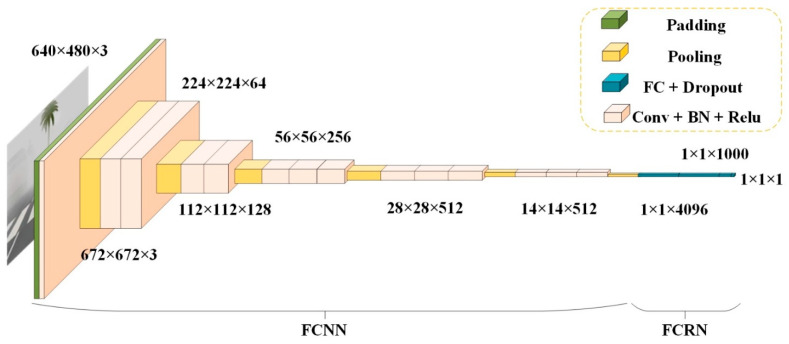
Structural diagram of the feature extraction and regression module (FERM).

The Feature Extraction Regression Module (FERM) is composed of a foggy convolutional neural network (FCCN) and a fully connected regression network (FCRN). On the whole, FERM is an improvement of VGG16 [39] for the visibility estimation task. First, FCNN is a feature extractor, and in this network, original image is first transformed into a 224 × 224 × 3 feature map by a padding layer, a convolution layer and a pooling layer to retain the local texture information of the fog image. As shown in Figure 3, the 2-nd-14th convolutional layers of the network adopt the same parameters as the first 13 convolutional layers of VGG16. After the 3rd, 5th, 8th, 11th, and 14th convolutional layers, a maximum pooling layer is added for down-sampling. In order to speed up training and prevent over-fitting, batch normalization (BN) [40] is conducted between each convolution layer and activation function, which can also prevent the proposed VISOR-NET from being sensitive to the initialization condition. Second, FCRN is a regressor consisting of four fully connected layers containing 4096, 4096, 1000 and 1 node in turn. In FCRN, dropout operation is added after the 1st–3rd fully connected layers to avoid over-fitting, and the coefficient is set to 0.1 uniformly. FCRN achieves regression prediction of fog level by returning a continuous value at the end.

The number of parameters in VGG16 and the FERM are approximately equal, but more image texture features can be selectively retained by our FERM. In Section 4.4, it is shown that the FERM is more suitable for visibility estimation from foggy images and achieves better performance that existing multi-classification algorithms [21].

### 3.3. Pairs Comparison with Relative Learning

Suppose there are M levels denoted by Y={1,…,M}. A set of labeled instances in level k can be denoted by $T^{k}=\{(x_{i}^{k},y_{i}^{k})|x_{i}^{k}\in X,y_{i}^{k}\in Y\}$. $\Phi (x_{i}^{k})$ is the visibility attributes of image $x_{i}^{k}$ extracted by the FCNN, and $f(\Phi (x_{i}^{k}))$ is the relative value of $x_{i}^{k}$ mapped by the FCRN. To simplify the discussion below, let us replace $(x_{i}^{k},y_{i}^{k})\in T^{k}$ with $\left(x^{k},y^{k}\right)$ to represent a labeled instance in level k.

According to the ordinal relationship, inter-class level labels $y^{k}$ satisfy the equation: $y^{1}<y^{2}<y^{3}\ldots <y^{M}$. Although the real visibility $v(x_{i}^{k})$ is unknown, the ordinal relationship of the relative value $f(\Phi (x_{i}^{k}))$ should also be consistent with the equation above, which can be formulated as:

(1) $$f(\Phi (x^{1}))<f(\Phi (x^{2}))<f(\Phi (x^{3}))\ldots <f(\Phi (x^{M}))$$

Via Equation (1), the proposed VISOR-NET adopts m tuple ordinal relations. The original relations are converted into a series of pairs:

(2) $$\begin{array}{l} f(\Phi (x^{1}))<f(\Phi (x^{2})),\;\ldots f(\Phi (x^{1}))<f(\Phi (x^{M})),\\ f(\Phi (x^{2}))<f(\Phi (x^{3})),\;\ldots \end{array}$$

This transformation can maintain the ordinal information between images. More importantly, it turns ordinal regression into relative learning, which can simplify the cost function and the learning process of the network. Considering a training batch $X_{batch}=\{x_{1},x_{2},\ldots ,x_{R}\}$ with corresponding label batch $Y_{batch}=\{y_{1},y_{2},\ldots ,y_{R}\}$, $R^{2}$ paired images can be obtained in every iteration. Because the intra-class relationship is unknown, all pairs with known ordinal relationships in one batch compose the paired training set $V=\{(x_{i},x_{j})|x_{i}\in X_{batch},x_{j}\in X_{batch},y_{i}\neq y_{j}\}$. An ordinal matrix $G=\{g_{ij}\}$ is defined to indicate the ordinal relative relationship of V.

(3) $$g_{ij}=\{\begin{array}{cc} 1, & y_{i}<y_{j}\\ 0, & y_{i}>y_{j} \end{array}{\;(x}_{i}{,x}_{j})\in V$$

Let $p_{ij}$ measure the relation between the output values f(Φ(.)) of paired images $\left(x_{i},x_{j}\right)$:

(4) $$p_{ij}=\frac{1}{1+\exp\{f(\Phi (x_{i}))-f(\Phi (x_{j}))\}}$$

where $o_{ij}=f(\Phi (x_{i}))-f(\Phi (x_{j}))$ is the visibility difference in pairs, and $p_{ij}$ is obtained by normalizing $o_{ij}$ with a sigmoid function. If $f(\Phi (x_{i}))\ll f(\Phi (x_{j}))$, $p_{ij}$ tends to 1; if $f(\Phi (x_{i}))\gg f(\Phi (x_{j}))$, $p_{ij}$ is close to 0. By using the sigmoid function, $p_{ij}$ is not sensitive when $o_{ij}$ exceeds the range of [−5, 5], which allows for a greater value of $o_{ij}$ for larger level differences in pairs. This means that the relative estimation of paired images is able to be extended to the entire dataset.

The logistic-like loss is adopted as the objective function:

(5) $$L_{s}=-\frac{1}{n}\sum_{(i,j)\in V}^{}\left(g_{ij}\log p_{ij}+(1-g_{ij})\log(1-p_{ij})\right)$$

where *n* is the number of paired images in sunset V.

### 3.4. Clustering Link

The network learns a regression function f(Φ(.)) within one batch by reducing $L_{s}$ to 0. When $p_{ij}$ tends to 1, according to (4), $o_{ij}$ tends to be larger and the difference value of pairs will increase gradually. However, too dispersed a value distribution is not conducive to prediction. Therefore, we add an optional clustering loss in (6) to limit the output range of the estimation value indirectly and make the inter-class distinction more obvious by reducing the intra-class distance:

(6) $$L_{c}=\frac{1}{2R}\sum_{k=1}^{M}\sum_{i=1}^{n^{k}}||\Phi (x_{i}^{k})-c^{k}|{|}_{2}^{2}$$

where $c^{k}$ is the central point of level k and should be updated during forward calculation in each iteration. $n^{k}$ is the number of images in level k of each batch. The composite loss function of the proposed VISOR-NET is defined in (7), where λ is a hyper parameter to balance $L_{s}$ and $L_{c}$.

(7) $$L=L_{s}+\lambda *L_{c}$$

### 3.5. Predicting Image Visibility Level

**Figure 4:**
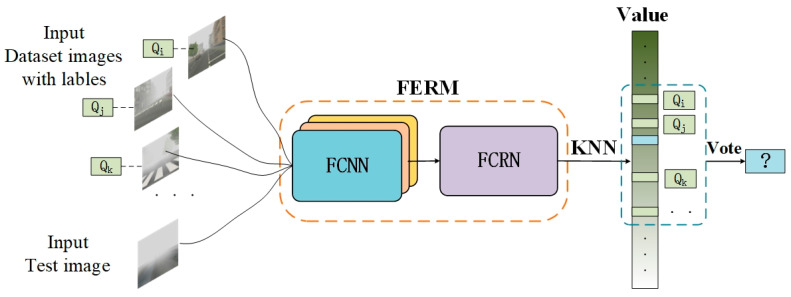
Schematic diagram of testing image visibility level.

Generally, in order to obtain classification results from a regression model, a series of thresholds—$\left\{S^{1},\ldots ,S^{k},S^{k+1};k=1,2,\ldots ,M\right\}$—needs to be set up in advance. The final classification result $y_{t}^{\prime }$ is determined by the location of the value $y_{t}^{\prime }=\{k|S^{k}<f(\Phi (x_{t}))<S^{k+1}\}$. Since the VISOR-NET pairs each image batch and cannot traverse all millions of pairs in the training dataset, there are still some instances in which the location is close to the boundary or even ventures into other categories. It is thus difficult to determine all the thresholds between the categories in our model. To solve this problem, we adopt the K-nearest neighbor (KNN) algorithm with majority voting to determine the final visibility level, which can help to avoid threshold tuning and to classify the hard samples more effectively. The demonstration diagram is shown in Figure 4.

Firstly, we create a query library Q by training set $T:Q=\{(f(\Phi (x_{i}),y_{i})|(x_{i},y_{i})\in T\}$. Then, in the prediction stage, we use the K nearest examples $x_{t}$. The prediction result $y_{t}^{\prime }$ is determined by the majority voting of $y_{i}$ in $N_{K}$:

(8) $$\begin{array}{l} y_{t}^{\prime }=\arg\max\sum_{x_{i}\in N_{K}}^{}I(y^{m},y_{i});\;m=1,2,\ldots M\\ s.t\;\;I(x,y)=\{\begin{array}{cc} 1, & if\;x=y\\ 0, & if\;x\neq y \end{array} \end{array}$$

### 3.6. Mapping a Relative Value to a Real Visibility

After the model is trained, we can obtain the relative visibility values of every image. However, it is difficult to evaluate the regression performance qualitatively using the relative output $\beta^{rel}$ of VISOR-NET. Therefore, we use some extra continuous labels as anchors to obtain a mapping function M(.). The relative values can be converted to absolute visibility values called Proposed (map). Because a real foggy image dataset with continuous visibility annotation is difficult to obtain, an INDF dataset with a continuous atmospheric extinction coefficient β is built based on atmospheric scattering model theory [41], which is a further development of Koschmieder’s law and explains the visibility principle of foggy images. The proposed method only uses discrete-level labeled data of the INDF dataset for training and obtains relative output $\beta^{rel}$. In order to compare the results of the proposed method and the regular regression method. After training, an extra 10% of continuous labels from training sets are selected as anchors. With a four-layer (32, 64, 32, 1) full connection network as a mapping function M(.), the absolute prediction values $\beta^{abs}$ of the remaining images are obtained from relative values $\beta^{rel}$. Specifically, the full connection network uses $\beta^{rel}$ as input, uses the corresponding real atmospheric extinction coefficient β as the true value, and uses the MES loss function, and finally linearly maps $\beta^{rel}$ to $\beta^{abs}$. Finally, we evaluate the regression effect of VISOR-NET using the absolute visibility prediction value. Specific experiments see Section 4.4.2.

## 4. Experiments and Discussions

Visibility estimation is an intermediate task between regression and classification, so the proposed VISOR-NET is evaluated in two ways: a classification task under discrete annotated datasets and a regression task under continuous annotated datasets.

### 4.1. Dataset

#### 4.1.1. Discrete Foggy Dataset

**Figure 5:**
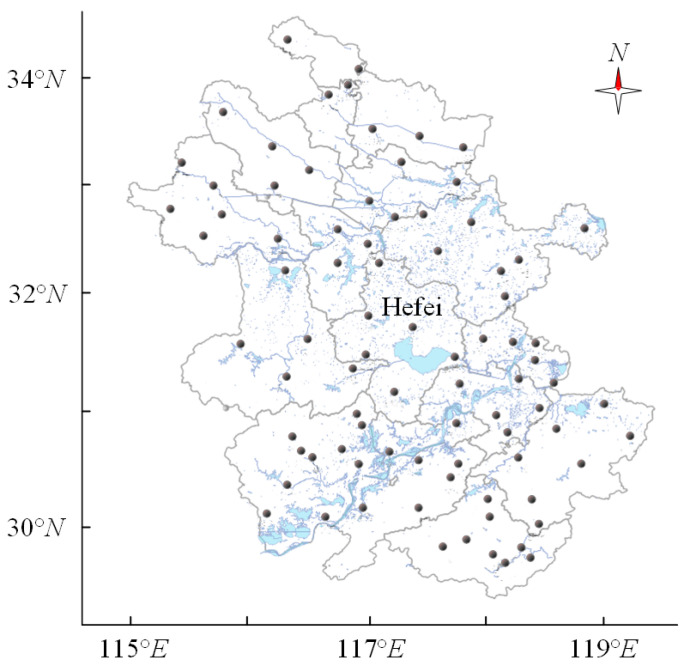
Map of cameras/stations (gray points) for the visibility observation.

FHVI dataset. Our dataset is collected from 84 highway-surveillance cameras located all over Anhui Province, China. The location of cameras for visibility observations can be seen in Figure 5. Every highway-surveillance camera is arranged to couple with a forward scattering visibility instrument at a meteorological station within 1 km (km). In spite of the visibility value provided by the meteorological department, we still faced many difficulties with annotation. Due to the unsteadiness of fog, the visibility value of different positions within 1 km varies greatly. Therefore, the obtained visibility value of the image is not very reliable. To make the annotation more accurate, we require the meteorological staff to use the visibility value as references to annotate the class label for each image. Falsely annotated images are removed.

The Grade of Fog Forecast from the China Meteorological Administration (GB/T 27964-2011) [42] classifies visibility below 1000 m into four classes, ranging from 1 to 4 (from dense fog to light fog), corresponding to 0–50 m, 50–200 m, 200–500 m and 500–1000 m, respectively. According to the advice of the Road Traffic Department, dense fog below 200 m has the greatest impact on traffic, and thus we add a level of 50–100 m between 50 and 200 m. Finally, we sifted through more than 30,000 images and obtained 5165 images that met the requirements. These images with a resolution of 640 × 480 are divided into six classes. The labels from 1 to 6 match visibilities of 0–50 m, 50–100 m, 100–200 m, 200–500 m, 500–1000 m, and 1000+ m, respectively. Since heavy fog is a rare weather phenomenon, the images of different classes are not evenly distributed.

FROSI dataset [16]. This is a standard set of synthetic images of simple road scenes for the evaluation of visibility estimators. In total, the dataset contains 3528 images with a resolution of 1400 × 600. For each image, a set of seven types of uniform fog is produced with visibility distances ranging from 50 m to 400 m.

ExFRIDA dataset [17]. This is a synthetic foggy image dataset that contains 3024 haze images from 84 various road scenes. All the images are generated by computer graphics software with a resolution of 640 × 480, and haze levels from 1 to 9 indicate fog from dense to light, but the visibility distance range of each level is not specified.

#### 4.1.2. Continuous Foggy Dataset

INDF dataset. This is a synthesized dataset with continuous labels based on the Indoor NYU-Depth dataset [43] and is generated to validate the proposed VISOR-NET for a dataset with continuous visibility labels. It is unavailable publicly. The atmospheric scattering model theory [41] in (9), which was developed using Koschmieder’s law, has been widely used for computer vision and building synthetic foggy-image datasets [44]. The hazy images H(x) can be generated by synthetic algorithms using the original images J(x) and depth map d(x):

(9) $$H(x)=J(x)e^{-\beta d(x)}+A(1-e^{-\beta d(x)})$$

where A is the atmospheric light that is set to a constant of 1.0. The atmospheric extinction coefficient β is also a constant based on the atmospheric uniformity hypothesis, where the higher the β, the lower the visibility of the synthesis image. A total of 4934 foggy images of 1000 indoor scenes are obtained at random β from 0.00 to 4.50, which are divided equally into nine levels where the labels from 9 to 1 match with β values of 0.00–0.50, 0.50–1.00, 1.00–1.50, 1.50–2.00, 2.00–2.50, 2.50–3.00, 3.00–3.50, 3.50–4.00, and 4.00–4.50, respectively.

**Figure 6:**
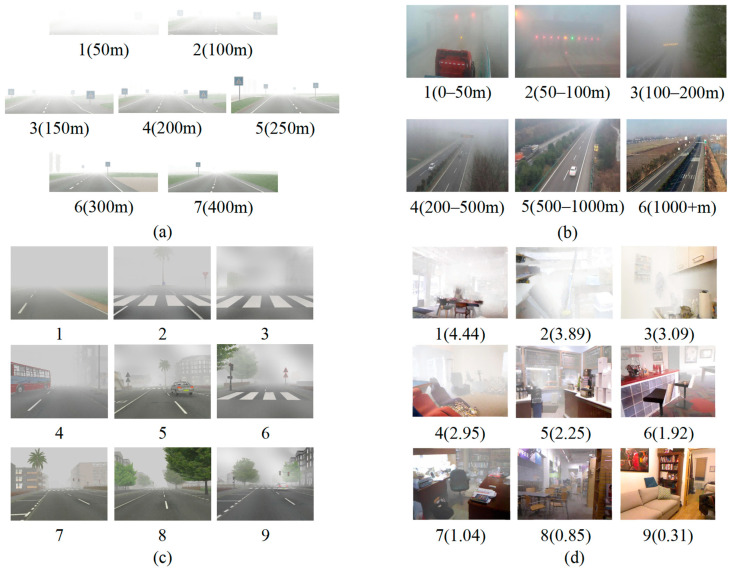
Images from the synthetic FROSI dataset with 7 haze levels (**a**), from the real FHVI dataset with 6 haze levels (**b**), from the ExFRIDA dataset with 9 haze levels (**c**), and from the INDF dataset with 9 haze levels (**d**).

Examples from these four datasets are shown in Figure 6. The details of four datasets are shown in Table 1, where class labels 1–9 represent the visibility level of the images; the higher the value, the higher the visibility level.

### 4.2. Baselines and Evaluation Index

#### 4.2.1. Classification Task

For the classification task, we introduce two baselines: a deep multi-classification algorithm [21] and an ordinal regression algorithm [14]. The first is a composition of a CNN with softmax logistic regression loss, which has been widely employed in many network architectures such as AlexNet [21], SCNN [22], VGG16 [39], and VisNet [23]. The proposed FERM network is also used in an ordinary classification task for comparison. This algorithm learns a probability density function p(.) to calculate the probability of a sample in all categories separately. The prediction $y^{\prime }$ is the index of the maximum probability p(x), which can be represented as $y^{\prime }=\arg\max(p(x),1)$.

The second type includes two deep ordinal regression algorithms described in Section 2.2 and the proposed VISOR-NET. All of them are first used in visibility estimation. One is named MOCNN [14], which converts ordinal regression to M−1 binary classification subproblems, and the final estimation is $\sum \begin{array}{c} M-1\\ k=1 \end{array}f_{cla}^{k}(x)+1$. The other is a threshold approaches CNNPOR [33], which adds a minimum margin constraint from adjacent ranks. We improved these ordinal regression algorithms for visibility estimation with the same FERM network in order to get an objective assessment.

Two evaluation criteria, i.e., classification accuracy (Acc) and mean absolute error (MAE), are used to evaluate the effectiveness of all methods. The classification accuracy is defined as $\sum \begin{array}{c} N\\ i=1 \end{array}[y_{i}=y_{i}^{\prime }]/N$, and MAE is defined as $\sum \begin{array}{c} N\\ i=1 \end{array}|y_{i}-y_{i}^{\prime }|/N$, where [.] is the truth-test operator, N is the total number of examples, and $y_{i}$ and $y_{i}^{\prime }$ are the ground truth and prediction of test image $x_{i}$, respectively.

#### 4.2.2. Regression Task

For the regression task, we use a regular regression algorithm with the FERM as the baseline under the INDF dataset, which maps foggy image x to ground truth value v with a regression function. Additionally, evaluation criteria Acc and MAE are used. The MAE is redefined as $\sum \begin{array}{c} N\\ i=1 \end{array}|v_{i}-v_{i}^{\prime }|/N$, where $v_{i}$ and $v_{i}^{\prime }$ are ground truth value and the estimated value of the testing image $x_{i}$, respectively. Cumulative accuracy (CA), another criterion, is defined by $\sum \begin{array}{c} N\\ i=1 \end{array}[|v_{i}-v_{i}^{\prime }|<c]/N$ and is also adopted to indicate the percentage of data whose absolute estimated error is smaller than the tolerance range c.

According to the actual needs of meteorological and traffic departments, absolute error within one level still meets the requirements of visibility warnings, traffic control, and other fields. So CA(1) criteria under the FHVI dataset alone is added to verify the practicability of the method in real application scenarios.

### 4.3. Implementation Details

In the FROSI and ExFRIDA datasets, 30% of the images are randomly selected as the test set and the remaining images are used as the training set. Due to the imbalance of our FHVI/INDF dataset, we randomly select 150/100 images from each level to make up a balanced testing set and the remaining images are used as the training set. To make the experimental results more objective, the experiment is repeated five times for each method, and the mean and variance are calculated for performance evaluation.

All the methods are implemented using Tensorflow and run on a NVIDIA 1080Ti Graphic Processing Unit (GPU). All networks are trained using ADAM solver with betas = (0.5, 0.999) and the batch size takes the maximum limit of the GPU, which is 48 in VGG/FERM/MOCNN/CNNPOR/VISOR-NET and 24 in AlexNet/SCNN/VisNet. The initial learning rate is set to 0.001 for the FHVI dataset and 0.0001 for the FROSI/ExFRIDA/INDF datasets with a linearly decay of 0.90 times per 5 epochs, and every experiment trained for 200 epochs.

### 4.4. Experimental Results and Discussions

#### 4.4.1. Classification Task

##### Hyper-Parameter Setting

There are two parameters, λ and k, in our VISORNET, and different combinations (λ,k) were tested in the model training to find the best value. The parameter *λ* is used for balancing $L_{s}$ and $L_{c}$, and *λ* dominates the intra-class variations. Different *λ* lead to different deep feature distributions. With proper *λ*, the discriminative power of deep features can be significantly enhanced. The experimental results are shown in Table 2 and Table 3. It can be seen that both parameters impact the final performance, and a lightweight λ with cluster loss $L_{c}$ has an obvious effect to the proposed VISOR-NET. The parameter *k* is a hyper parameter in the K-nearest neighbor (KNN) algorithm. Choosing the optimal *k* value is a necessary condition to build a reasonable and accurate KNN model. If the value of *k* is too low, the model will become too specific and the generalization performance will become worse. On the contrary, if the value of *k* is too high, the model will become over-generalized, resulting in under-fitting. However, as shown in Table 2 and Table 3, the influence of k is not very strong, and the performance shows little variation with different values of k when λ = 0.001. Therefore, in order to reduce the amount of computation, we choose *k* = 5 in our experiments.

##### Comparisons and Discussions

To calculate the contribution of our improved FERM module and the ordinal relative learning algorithm, two kinds of comparison experiments were conducted. Firstly, the different network structures of the multi-classification algorithms Alexnet, SCNN, VGG16, VisNet, and FERM were compared. Secondly, the VISOR-NET was compared with three baseline algorithms, i.e., FERM, MOCNN, and CNNPOR, which all use FERM as the basic network structure.

The best parameter combination λ=0.001,k=5 was selected to compare with the baselines under three discrete foggy datasets with class labels, and the results are shown in Table 4. Bold numbers in the table represent the best performance. Due to the simple scenes and the obvious differences between levels in the FROSI dataset, the accuracy of all methods was close to 100%. It can be seen from Table 4 that the FERM achieved the best performance the in multi-classification task, which proves that our improved FERM network is more reasonable for the visibility task. Three deep ordinal regression algorithms MOCNN, CNNPOR, and our proposed method all surpass the results of the multi-classification algorithm, even under the same FERM CNN structure, which means the ordinal information in foggy images is helpful for visibility estimation.

The experimental results also indicate that the proposed ordinal relative learning is more effective than the two ordinal regression algorithms MOCNN and CNNPOR. The practicable criteria CA(1) in the real application scenarios in the FHVI dataset were nearly 100% under the proposed VISOR-NET, which meets the visibility estimation requirements for practical applications.

**Figure 7:**
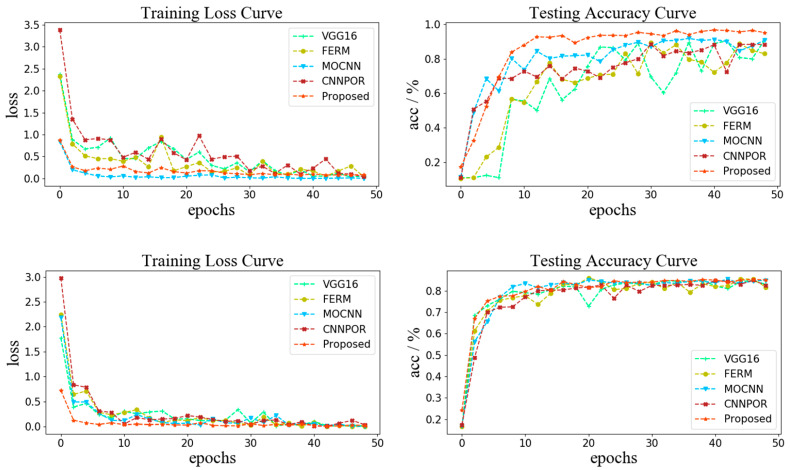
The upper part shows the training loss and testing accuracy with the ExFRIDA dataset, and the lower part shows the training loss and testing accuracy with the FHVI dataset.

Figure 7 shows the details of the training loss and testing accuracy for the proposed VISOR-NET and four methods (VGG16, FERM, MOCNN, CNNPOR). It is clear that the convergence process of our method is more rapid and stable than that of other baselines, and it is similar in its testing accuracy curve. Therefore, the proposed method outperforms the other baselines in terms of model stability and prediction accuracy.

**Figure 8:**
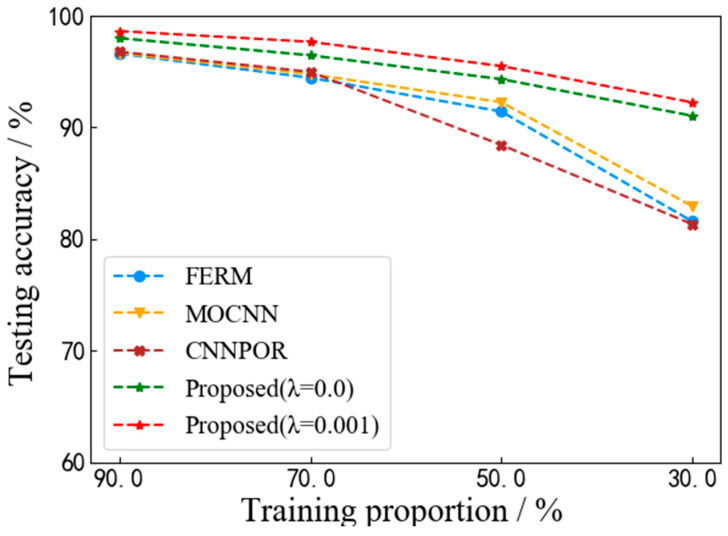
Acc curves with different proportions of training sets from the ExFRIDA dataset.

Since images with accurate visibility labels are difficult to obtain in real applications, we perform comparison experiments with different proportions of training sets from the ExFRIDA dataset. The accuracy curves in Figure 8 show that the proposed VISOR-NET can still achieve competitive performance when the number of training data decrease from 90% to 30%, which is important for real scenes, especially when there are not many annotated data available.

We also looked at the consumption of computing resources of the main methods. Table 5 shows the run time per iteration or per epoch for the ExFRIDA dataset, which can reflect the computing-resource consumption of the methods. They are all based on the same FERM with the same GPU: 1080Ti, and the batch size in each is 48. The VISORNET algorithm does not to use all paired images directly and the input method of images to the network in one batch is similar that of other normal classification baselines. After one batch of images is input to the network, the features of every image are extracted and the relative visibility value is calculated by VISOR-NET. Then, thousands of pairs in one batch are formed and the relative loss is calculated from the paired relative visibility. In deep-learning methods, the main consumption of computing resources comes from the feature extraction and backpropagation. There is no difference between the VISOR-NET and baselines under the same basic network. Compared with other deep classification baselines, the total consumption of computational resources is not increased by much. The experimental results show that the increased computational resources are acceptable if considering the improved classification performance.

##### Results at Different Levels

The prediction accuracy of two datasets (FROSI/ExFRIDA) at each level is shown in Table 6 and Table 7. Bold numbers in the table represent the best performance. The accuracy of all methods at a low visibility level is significantly lower than at high visibility, which means that visibility estimation in dense fog is more difficult than under light fog under learning-based methods. Compared with the images of light fog, the dense foggy images have fewer structural features and the difference in visibility characteristics between adjacent ranks is smaller. This shows that the proposed VISOR-NET algorithm can achieve much better performance for low-visibility images, such as class 1–5 in the ExFRIDA dataset and 0–100 m in the FHVI dataset. The high accuracy of the proposed VISOR-NET algorithm in low visibility circumstances shows that our method can utilize the comparative visibility features of foggy images and, therefore, improves the estimation effect for dense foggy images.

#### 4.4.2. Regression Task

**Figure 9:**
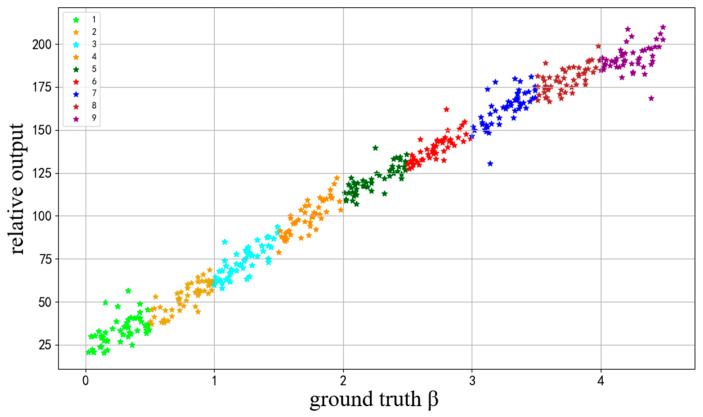
The relative visibility prediction $\beta^{rel}$ of VISORNET and the ground truth β with the IDNF dataset, where 50 test examples are randomly selected from each category.

In order to evaluate the regression estimation effect of the proposed VISOR-NET algorithm, we carried out experiments on the INDF dataset with continuous labels. Figure 9 represents some test samples of the relative outputs $\beta^{rel}$ with the corresponding ground truth β. The experimental results show that the proposed VISOR-NET algorithm can learn a global rank function effectively. This means that the relative output values can be linearly mapped to real visibility since the ordinal ranks are preserved. Moreover, it indicates that relative ordinal learning can quantify the visibility difference between images and obtain the approximate estimation of visibility without continuous labels. It also shows that the better classification results of VISOR-NET are due to the more reasonable learning process. A further qualitative discussion of the visibility estimation will be presented in the subsequent regression experiments.

#### Comparisons and Discussions

After adding 10% more continuous labels to the training set, the relative outputs $\beta^{rel}$ of the VISOR-NET can be mapped to the absolute values $\beta^{abs}$ by a simple four-layer fully connected neural network. Table 8 shows the regression results of the Proposed (map) and regular regression with the same CNN structure and the classification result of the FERM and the proposed algorithm with the INDF dataset. Compared with the discrete prediction results of the classification task, the output of the regression task is linearly continuous, so the MAE index of the Proposed (map) and regular regression is smaller. In addition, since all continuous labels of the training set are added, the regression effect of the regular regression algorithm is satisfied. While the Proposed (map) only uses a small number of continuous labels (10%) in the mapping stage, and the whole ordinal regression learning process is only dependent on discrete-level labels, the final regression effect is weaker than regular regression, and it is approximate to the regular regression effect when the absolute error of the CA is larger than 0.2.

#### Results at Different Levels

**Figure 10:**
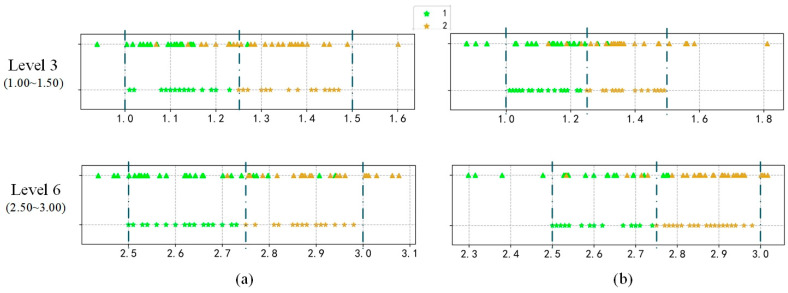
The distribution of 50 randomly sampled values in levels 3/6 from the INDF dataset, where the star pattern ‘⋆’ represents the ground truth and the triangle pattern ‘▲’ represents the prediction value in each level, with the regression method (**a**) or VISOR-NET (**b**).

To further show that VISOR-NET retains the intra-class difference and realizes the continuous estimation of foggy images, we annotate the distribution of predicted value $\beta^{abs}$ and the real value β of foggy images within one class, and the results are shown in Figure 10. It can be seen that VISOR-NET realizes the binary classification within one class, which indicates that the proposed VISORNET can learn not only the rank from the discrete level but also the global estimation function for all data.

We also analyzed the regression results at each level with the regression criteria CA (0.15) and MAE (Table 9), which shows that our method exhibits better performance in the middle levels (2–8) than in the first or last level. The same phenomenon can also be seen in Figure 9, in which the distribution of relative values in the 1st and 9th levels are more diffuse. A possible reason for this is that the images at the beginning or end only accept one relative constraint, such as $f(x^{1})<f(x^{k})$ in level 1 and $f(x^{M})>f(x^{k})$ in level M, while in the middle levels k have intact relative constraints such as $f(x^{k-1})<f(x^{k})<f(x^{k+1})$. Since the relative estimation results of the first and last level are limited by the lack of boundary constraint conditions, the proposed VISOR-NET cannot achieve a satisfying regression result—although it also works well for rough level estimation.

## 5. Conclusions

This paper proposed VISOR-NET, a novel end-to-end pipeline that uses the ordinal information and relative relation of the images to guide visibility estimation. To the best our knowledge, it is the first time an ordinal regression model has been used to estimate visibility under discrete-level labels. Since there is no visibility dataset of real images that is publicly available, we collected a large-scale dataset, FHVI, taken from real surveillance scenes. To evaluate the VISOR-NET algorithm’s performance, we compared VISOR-NET with other state-of-the-art deep learning-based methods by carrying out experiments with three datasets (FROSI, ExFRIDA, and FHVI). The extensive experimental results demonstrate that the proposed VISOR-NET algorithm is more effective than others, and the convergence analysis also shows that VISOR-NET is more stable and requires fewer examples during the training stage. Moreover, we synthesized an INDF image dataset with continuous labels to analyze the global estimation effectiveness of the relative output, which indicated that VISOR-NET has the potential ability to map real visibility values using only a few anchor images. Moreover, the proposed solution is applicable to the existence of ordinal relationships between different classes, which can make full use of the ordinal information in the data to achieve more accurate feature extraction and estimation.

## Figures and Tables

**Table 1:** The distribution of four datasets with detailed levels and quantities.

Datasets	1	2	3	4	5	6	7	8	9	Total
FROSI	504	504	504	504	504	504	504	/	/	3528
ExFRIDA	336	336	336	336	336	336	336	336	336	3024
FHVI	308	625	774	893	951	1614	/	/	/	5165
INDF	678	618	554	529	531	557	566	558	543	4934

**Table 2:** The mean Acc and MAE of VISOR-NET under different hyper parameters with the ExFRIDA dataset.

Hypers	*λ* = 0.0		*λ* = 0.001		*λ* = 0.01		*λ* = 0.1	
	Acc	MAE	Acc	MAE	Acc	MAE	Acc	MAE
*k* = 5	94.66%	0.0533	97.53%	0.0245	95.97%	0.0408	95.86%	0.0414
*k* = 25	94.80%	0.0519	97.47%	0.0253	95.94%	0.0408	95.92%	0.0408
*k* = 50	95.03%	0.0522	97.55%	0.0244	96.00%	0.0403	95.97%	0.0381

**Table 3:** The mean Acc and MAE of VISOR-NET under different hyper parameters with the FHVI dataset.

Hypers	*λ* = 0.0		*λ* = 0.001		*λ* = 0.01		*λ* = 0.1	
	Acc	MAE	Acc	MAE	Acc	MAE	Acc	MAE
*k* = 5	86.67%	0.1378	87.83%	0.12723	87.33%	0.1400	86.17%	0.148
*k* = 25	86.06%	0.1439	87.72%	0.1289	87.11%	0.1422	85.67%	0.1528
*k* = 50	85.67%	0.1472	87.76%	0.1284	87.06%	0.1428	85.61%	0.1533

**Table 4:** Experimental results of different deep-learning methods with three datasets.

Method	FROSI		ExFRIDA		FHVI		
	Acc	MAE	Acc	MAE	Acc	MAE	CA(1)
Alexnet	98.27	0.018	70.41 ± 1.21	0.323 ± 0.009	69.41 ± 0.61	0.344 ± 0.011	97.58 ± 0.15
SCNN	98.76	0.015	62.86 ± 1.29	0.412 ± 0.018	70.55 ± 0.45	0.363 ± 0.012	96.64 ± 0.54
VGG16	98.60	0.014	86.97 ± 0.41	0.144 ± 0.004	84.16 ± 0.52	0.179 ± 0.007	98.02 ± 0.05
VisNet	98.40	0.017	88.08 ± 0.82	0.114 ± 0.006	83.64 ± 0.65	0.181 ± 0.016	98.27 ± 0.15
FERM	100	0.0	94.09 ± 0.84	0.059 ± 0.009	85.89 ± 0.26	0.162 ± 0.004	98.16 ± 0.09
MOCNN	100	0.0	94.65 ± 0.75	0.052 ± 0.007	86.44 ± 0.56	0.150 ± 0.007	98.72 ± 0.08
CNNPOR	100	0.0	94.68 ± 0.63	0.049 ± 0.008	86.05 ± 0.32	0.102 ± 0.007	99.16 ± 0.16
Proposed	100	0.0	**97.55 ± 0.35**	**0.024 ± 0.003**	**87.83 ± 0.55**	**0.127 ± 0.006**	**99.78 ± 0.05**

**Table 5:** The run time per iteration or per epoch for the proposed and baseline algorithms with the ExFRIDA dataset.

Method	Run Time/Iteration	Run Time/Epoch
VGG16	0.955 s	40.465 s
FERM	1.130 s	47.465 s
MOCNN	1.072 s	45.037 s
CNNROP	1.169 s	49.129 s
VISOR (*λ* = 0.0)	1.172 s	49.323 s
VISOR (*λ* = 0.001)	1.177 s	49.437 s

**Table 6:** Experimental results of different deep methods at each level with the ExFRIDA dataset.

Visibility (%)	Class 1	Class 2	Class 3	Class 4	Class 5	Class 6	Class 7	Class 8	Class 9
Alexnet	64.0	44.0	57.0	58.5	70.0	76.0	79.0	86.0	93.5
SCNN	53.5	28.5	44.5	57.5	43.5	54.0	65.5	82.5	93.5
VGG16	72.0	75.0	83.0	86.5	87.5	89.5	94.0	98.5	**100**
VisNet	67.0	81.5	80.5	85.5	87.5	91.5	94.5	96.0	98.5
FERM	79.3	91.7	94.0	95.0	95.7	96.0	99.0	**100**	99.7
MOCNN	90.0	81.7	93.3	92.8	96.3	99.0	**99.3**	99.7	99.7
CNNPOR	85.2	85.8	93.3	95.7	95.2	98.0	99.0	99.5	99.8
Proposed	**91.3**	**93.0**	**95.0**	**98.0**	**98.7**	**99.7**	**99.3**	**100**	**100**

**Table 7:** Experimental results of different deep methods at each level with the FHVI dataset.

Visibility (%)	0–50 m	50–100 m	100–200 m	200–500 m	500–1000 m	1000+ m
Alexnet	0.0	80.3	75.6	79.3	83.8	97.6
SCNN	1.3	75.3	**84.6**	75.3	89.3	98.0
VGG16	73.7	78.0	79.0	82.0	90.7	99.0
VisNet	75.3	74.0	79.0	**84.2**	90.7	99.3
FERM	84.4	77.3	82.2	82.4	90.4	98.7
MOCNN	84.7	80.9	79.1	82.9	93.1	98.2
CNNPOR	80.0	81.5	84.2	80.3	91.2	98.5
Proposed	**85.5**	**83.6**	81.8	**84.2**	**94.2**	**100**

**Table 8:** The experimental results of different methods with the INDF dataset.

Visibility (%)	Acc (%)	MAE	CA (0.05)	CA (0.1)	CA (0.2)	CA (0.3)	CA (0.4)
FERM	74.22	0.2322	/	/	/	/	/
Proposed	80.50	0.1944	/	/	/	/	/
Proposed (map)	79.78	0.1184	29.00	55.00	82.55	93.22	97.89
Regression	88.11	0.0768	58.55	87.20	98.43	99.68	99.95

**Table 9:** The regression results at each level for two methods.

Visibility (%)		1	2	3	4	5	6	7	8	9
Regression	CA(0.15)	91.8	83.8	95.4	98.0	98.3	98.8	98.8	100	98.9
	MAE	0.0698	0.0609	0.0863	0.0471	0.0429	0.0398	0.0394	0.0400	0.0439
Proposed (map)	CA(0.15)	34.0	70,0	76.0	84.0	92.0	80.0	81.0	90.0	53.0
	MAE	0.2373	0.1073	0.1236	0.0906	0.0616	0.0950	0.0948	0.0807	0.1742

## Data Availability

The data used to support the findings of this study are available from the corresponding author upon request.
